# Cross-Seeding of Misfolded Proteins: Implications for Etiology and Pathogenesis of Protein Misfolding Diseases

**DOI:** 10.1371/journal.ppat.1003537

**Published:** 2013-09-19

**Authors:** Rodrigo Morales, Ines Moreno-Gonzalez, Claudio Soto

**Affiliations:** Mitchell Center for Alzheimer's Disease and Related Brain Disorders, Department of Neurology, University of Texas Houston Medical School, Houston, Texas, United States of America; Washington University School of Medicine, United States of America

## Introduction

Accumulation of misfolded protein aggregates is a hallmark event in diverse diseases. These structures are able to seed their own polymerization by acting as aggregation nuclei both *in vitro* and *in vivo*. Recent studies in animal models suggest that misfolded proteins associated with different diseases can synergize in a heterologous fashion, potentiating pathological mechanisms and accelerating disease progression. The coexistence of misfolded protein aggregates has been described in patients affected by several protein misfolding disorders, suggesting a possible molecular cross-talk between pathological processes associated with different diseases. One putative mechanism for this cross-talk is a direct interaction between misfolded proteins, leading to *cross-seeding* of protein aggregation. This article summarizes the evidence for the cross-seeding phenomenon recently obtained in studies performed *in vitro*, in animal models, and in human patients, as well as the potential contribution of this mechanism to our understanding of the still elusive etiology and progression of maladies such as Alzheimer's disease, where no effective diagnostic or therapeutic strategies exist.

## Misfolded Proteins, Amyloids, and the Seeding-Nucleation Model of Aggregation

**Figure 1:**
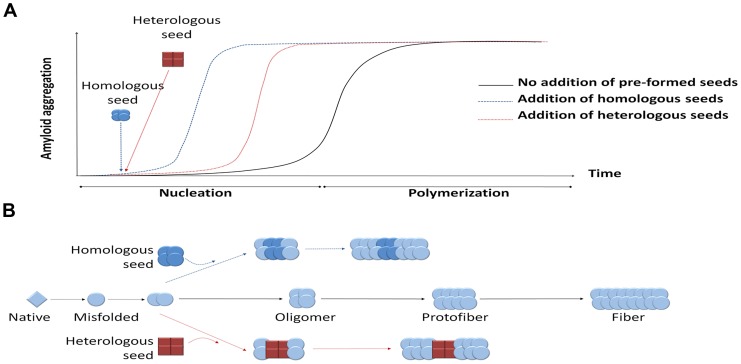
Seeding-nucleation model of protein aggregation and the cross-seeding phenomenon. (A) Amyloid aggregates are formed following the seeding-nucleation polymerization model. This aggregation process is divided into two phases, the so-called nucleation/lag phase and the polymerization/elongation phase (solid lines). Since nuclei are formed, the aggregation increases in an exponential manner from small oligomers to fibers. The addition of preformed seeds leads to a shorter lag phase and a faster aggregation (dashed lines). (B) Seeding can occur by adding a previously formed seed, facilitating and speeding up the polymerization process. These seeds can have the same chemical nature as the nuclei, leading to a homologous seeding, or be made from a different protein, inducing a heterologous seeding or cross-seeding.

Disease-associated misfolded proteins usually organize in β-sheet conformations that tend to form large aggregates and accumulate in different organs in the form of amyloid deposits, becoming toxic and leading to diverse protein misfolding diseases (PMDs), such as Alzheimer's disease (AD), transmissible spongiform encephalopathies (TSEs), Parkinson's disease (PD), and type 2 diabetes (T2D), among many others [1]. The seeding-nucleation polymerization model explains how amyloid aggregates are formed (Figure 1) [2]. Oligomeric, misfolded protein “seeds” are thought to be produced during the nucleation or lag phase, a thermodynamically unfavorable process involving various intermediates including partially denatured or mutated monomeric proteins as well as small unstable oligomers of diverse size [2], [3]. During the second step of this process, termed polymerization or exponential phase, these seeds induce a fast and exponential recruitment of the normally folded monomeric protein. As a result, a wide variety of misfolded structures are formed, ranging from small soluble oligomers to large fibrillar deposits. *In vitro* studies show that the additional new seeds act as templates for further polymerization, reducing the length of the lag phase (Figure 1, panel A) [2]–[4]. This seeding process could be homologous or heterologous (Figure 1, panel B) [5]. Homologous seeding has been widely documented for many of the proteins implicated in PMDs [5]. Seeding is likely the basis by which the misfolded prion protein acts as an infectious agent to propagate prion diseases [3], [6]. Interestingly, several recent exciting reports have shown that under defined experimental conditions, various other misfolded proteins can spread the pathology between cells and tissues and even induce the disease features when administered into animal models [6]–[10].

Heterologous seeding, also known as “cross-seeding,” occurs when oligomers composed by one misfolded protein can promote the polymerization of a different protein [5]. Cross-seeding processes may provide a mechanistic explanation for various observations in distinct diseases, including: i) the simultaneous presence of different misfolded proteins in one disease; ii) the coexistence of more than one PMD in the same individual; iii) the epidemiological observation that one PMD may be a risk factor for development of a second PMD; and iv) the exacerbation of clinical features when various misfolded protein aggregates accumulate simultaneously. Furthermore, since many proteins form amyloid-like misfolded aggregates as part of their normal biological function [6], cross-seeding with functional amyloids may play an important and yet uncovered role in the origin of PMDs.

## Cross-Seeding between Misfolded Proteins: The *In Vitro* Evidence

The direct interaction of misfolded proteins, a subject poorly explored so far, could play a major role in the genesis and progression of several pathological conditions. Although, not extensively studied, there are various reports showing cross-seeding interaction among several amyloidogenic proteins *in vitro* [11]–[17]. Some studies have suggested that the ability of certain proteins to cross-seed may depend upon the specific conformation that the misfolded seed acquires, but also on the sequence/conformation of the monomeric-soluble protein to be seeded. Experiments using hen lysozyme showed that the capability of this protein to be seeded was directly proportional to the sequence homology with the misfolded seed [11]. However, experiments in the test tube demonstrated that amyloidogenic proteins substantially differing in their sequences were able to interact and potentiate their aggregation processes, albeit with a lower efficiency than the homologous protein [5], [12]. Due to its capital involvement in AD pathogenesis, one of the most studied proteins in cross-seeding experiments is Amyloid-β (Aβ). Several studies have documented an interaction between Aβ and other misfolded proteins, including prion protein [13], tau [14], and α-synuclein [15]–[17]. From these studies an important feature is revealed: the interaction between amyloidogenic proteins may work in both directions. For example, Aβ aggregates can seed the polymerization of prions and α-synuclein, and aggregates of those proteins can also accelerate the polymerization rate of soluble Aβ. Similar findings have been observed for other proteins implicated in PMDs [5]. However, sometimes the cross-seeding effect is unidirectional, and other times the interaction results in cross-inhibition of protein aggregation. An example of the former involves the interaction between Aβ and Islet Amyloid Polypeptide (IAPP, a protein that forms amyloid aggregates in the pancreas and is involved in T2D). *In vitro* results showed that Aβ acts as a good seed on IAPP polymerization; however, IAPP aggregates have little or no effect over soluble Aβ oligomerization [12]. A more complex result was reported for the interaction between apolipoprotein A2 and serum amyloid A; depending upon the experimental conditions, these proteins can both cross-seed and cross-inhibit amyloid formation [18].

## Interaction of Pathogenic Proteins in Animal Models

Although the putative biological consequences of cross-seeding have not been investigated in detail, there are several studies suggesting a molecular cross-talk between misfolded proteins *in vivo*. Perhaps the most emblematic study of cross-seeding in animal models is the one involving Aβ and tau proteins. The simultaneous brain accumulation of these proteins is the main hallmark of AD. Studies in animal models using various transgenic mice revealed that Aβ is able to accelerate the aggregation of tau; however, it seems that tau aggregates do not have the same effect over Aβ [19], [20]. These findings suggest a possibly unidirectional cross-seeding. However, it is also possible that this outcome may be mediated by indirect processes, for example, Aβ aggregates may activate certain kinases responsible for tau phosphorylation, leading to higher misfolding and aggregation of this protein [21].

Another interaction that has been studied in some detail is between α-synuclein and the misfolded proteins associated with AD. The presence of α-synuclein aggregates has been observed in approximately 50% of the AD patients, and this has been associated with more severe pathological outcomes [22]. Transgenic mice developing both Aβ and α-synuclein aggregates in their brains recapitulate some of the pathological features observed in these patients, including accelerated cognitive decline, and higher accumulation of misfolded aggregates compared to single transgenics [23]. *In vitro* studies involving pure α-synuclein and Aβ support the interaction between these proteins [17]. Experiments in murine models combining α-synuclein, Aβ, and tau showed that these animals display an accelerated cognitive decline as measured by loss of spatial memory [16]. As expected, this is positively correlated with the deposition of all three amyloidogenic proteins.

Another study analyzing the cross-seeding effect between misfolded proteins was done at our lab taking Aβ and infectious prions (PrP^Sc^) as models [13]. The leading role of Aβ in AD, along with the clear phenotype of prion disease in animal models (clinical signs followed by death), led us to analyze the extent of interaction of these proteins in an *in vivo* situation. After intraperitoneal injection of RML prions in transgenic models of AD, we observed a significant acceleration of the prion pathology compared to wild-type littermates injected with the same agent [13]. The acceleration of the disease phenotype was dependent on the amount of Aβ aggregated in the brain. Interestingly, the deposition of Aβ plaques was higher in animals injected with prions compared to the ones receiving just buffer, suggesting that the presence of misfolded prions is also able to enhance AD features. Moreover, we found localized presence of prion protein within Aβ aggregates in addition to the diffuse PrP aggregation typically found in wild-type animals injected with this particular prion strain. These findings were complemented by cell-free assays showing a bidirectional interaction of prions and Aβ in terms of seeding and aggregation [13].

An interesting earlier study by Westermark and colleagues showed convincing evidence for exogenous induction of pathology through cross-seeding in a mouse model of AA amyloidosis [24]. In this model, deposition of AA amyloid develops spontaneously after subjecting the animals to an inflammatory challenge. The time for appearance of deposits can be shortened dramatically by administration of a small amount of tissue extract containing amyloid aggregates from a sick animal. Intravenous injection of preformed amyloid fibrils made from synthetic peptides corresponding to parts of several different misfolded proteins resulted also in acceleration of AA amyloid accumulation [24]. Remarkably, using radiolabeled heterologous, synthetic fibrils, these authors found that the injected materials deposited in various organs and that new AA-amyloid fibrils developed onto the exogenously administered seeds. The coexistence of the two heterologous proteins in the same fibrillar aggregate was confirmed by immunogold electron microscopy studies of the tissue [24]. These results provide direct evidence for cross-seeding as a putative disease mechanism.

## Evidence of Cross-Talk between Protein Misfolding Disorders in Human Studies

The fact that misfolded proteins can interact and accelerate their rate of aggregation *in vitro* and in animal models suggests that the simultaneous occurrence of different misfolded proteins may enhance the onset and progression of certain pathological conditions. Remarkably, several epidemiological studies support this idea. One interesting and well-supported case is the interaction between AD and T2D. It has been described that about 81% of AD patients are also affected by either T2D or impaired fasting glucose [7]. In addition, and comparing with age- matched nondemented individuals, AD patients show a higher incidence of islet amyloidosis than healthy individuals. Conversely, multiple epidemiological studies have shown that T2D patients exhibit an increased risk of developing AD compared with nondiabetic individuals matched for age and sex (for reviews see [25], [26]). However, it is important to highlight that the linkage between these two diseases has not always been observed or in some studies occurs in only one direction. The mechanism for the risk association between AD and T2D is unknown and several hypotheses have been proposed, including: alterations in insulin signaling, oxidative stress, abnormal clearance capacity, hypercholesterolemia, and interactions at the level of protein misfolding [5], [27]. Although the presence of misfolded aggregates at different organs throughout the body complicates the possibility of a cross-seeding in this specific case, it is important to highlight that there is evidence for soluble oligomers circulating in biological fluids [28], [29].

Protein aggregates associated with various diseases often show the presence of other misfolded proteins within the deposits, as determined by immunostaining and co-immunoprecipitation analyses. For example, amyloid plaques in several cases of AD have been reported to contain aggregated PrP [30], [31]. Similar observations have been made in patients affected with either sporadic Creutzfeldt-Jakob disease or Gerstmann-Sträussler-Scheinker syndrome, where the presence of Aβ within the typical prion plaques observed in these diseases is clear [32], [33]. Maybe more important is the case of patients affected with the Lewy-body variant of AD [22]. Patients suffering from this “triple brain amyloidosis” show a more aggressive disease progression compared to individuals affected by classical AD. As mentioned earlier, the interaction of Aβ, α-synuclein, and tau has been also studied *in vitro* and in animal models.

## Cross-Seeding and Alternative Pathways to the Cross-Talk between Misfolded Proteins

Although a direct interaction between misfolded proteins through ordered events of cooperative misfolding is appealing and is supported by *in vitro* experiments, there are various other alternative explanations for the synergistic interaction between diverse PMDs observed in animal models and human studies. The alternative pathways to the cross-seeding mechanism include among others: enhancement of cellular vulnerability, impairments in clearance machinery, excessive tissue inflammation, increase of oxidative stress, and triggering of other signal transduction pathways resulting in increase of protein misfolding. Among them, the possibility that the simultaneous presence of various protein aggregates produce defects in clearance mechanisms seems quite feasible. It is well established that the net accumulation of misfolded protein aggregates depends on the relative balance between the rate of formation of new aggregates and their removal [34], [35]. It is likely that the clearance machinery already impaired by one misfolded protein may be further diminished by a second misfolding event, leading to the faster and higher accumulation of both type of aggregates and the subsequent cellular and tissue damage [13], [36].

Exploring further the molecular cross-talk between PMDs and its underlying mechanism seems essential to understand how these complex processes participate in the origin and progression of PMDs. It is important to highlight that a large number of the PMDs cases are etiologically classified as “sporadic.” The term sporadic is used since no clear cause for the pathogenesis is known. In order to explain the sporadic origin of these diseases, several hypothesis have been proposed, mostly arguing that various processes act as risk factors, including aging, inflammation, oxidative stress, and environmental factors, etc. [37]. In addition, genetic predisposition by mutation or polymorphism in accessory proteins has been identified [38]. All these events, although well characterized, do not account for the majority of sporadic cases. The cross-seeding phenomenon could contribute to explaining, at least in part, the still unknown origin of several of these pathological conditions.
